# Trends in influenza vaccination coverage in Portugal from 1998 to 2010: effect of major pandemic threats

**DOI:** 10.1186/1471-2458-13-1130

**Published:** 2013-12-05

**Authors:** Cátia Sousa Pinto, Baltazar Nunes, Maria João Branco, José Marinho Falcão

**Affiliations:** 1 Public Health Unit, North Lisbon Health Care Center, Largo Professor Arnaldo Sampaio, Lisbon, Portugal; 2 Department of Epidemiology, National Institute of Health Doutor Ricardo Jorge, Avenida Padre Cruz, Lisbon, Portugal

**Keywords:** Influenza, Vaccine coverage, Vaccination trends

## Abstract

**Background:**

Vaccination is the key measure available for prevention of the public health burden of annual influenza epidemics. This article describes national trends in seasonal influenza vaccine (IV) coverage in Portugal from 1998/99 to 2010/11, analyzes progress towards meeting WHO 2010 coverage goals, and addresses the effect of major public health threats of the last 12 years (SARS in 2003/04, influenza A (H5N1) in 2005/06, and the influenza A (H1N1)2009 pandemic) on vaccination trends.

**Methods:**

The National Institute of Health surveyed (12 times) a random sample of Portuguese families. IV coverage was estimated and was adjusted for age distribution and country region. Independence of age and sex coverage distribution was tested using a modified F-statistic with a 5% significance level. The effect of SARS, A (H5N1), and the A (H1N1)2009 pandemic was tested using a meta-regression model. The model was adjusted for IV coverage in the general population and in the age groups.

**Results:**

Between 1998/99 and 2010/11 IV, coverage in the general population varied between 14.2% (CI _95%_: 11.6%–16.8%) and 17.5% (CI _95%_: 17.6%–21.6%). There was no trend in coverage (p = 0.097). In the younger age group (<15 years) a declining trend was identified until 2008/09 (p = 0.005). This trend reversed in 2009/10. There was also a gradual and significant increase in seasonal IV coverage in the elderly (p for trend < 0.001). After 2006/07, IV coverage remained near 50%. Adjusting for baseline trends, there was significantly higher coverage in the general population in 2003/04 (p = 0.032) and 2005/06 (p = 0.018). The high coverage observed in the <15-year age group in season 2009/10 was also significant (p = 0.015).

**Conclusions:**

IV coverage in the elderly population displayed an increasing trend, but the 75% WHO 2010 target was not met. This result indicates that influenza vaccination strategy should be improved to meet the ambitious WHO coverage goals. The major pandemic threats of the past decade had a modest but significant effect on seasonal influenza vaccination. There was an increase in vaccine uptake proportion in the general population in 2003/04 and in 2005/06, and in individuals <15 years old in 2009/10.

## Background

The public health burden of annual influenza epidemics represents ongoing vulnerability to pandemic influenza and highlights gaps in bioterrorism preparedness and response efforts [1,2]. The recent emergence of the pandemic influenza A (H1N1) 2009 virus is a good example of how influenza can impact health systems around the world [3].

Currently, vaccination is the key measure available for prevention of influenza and associated complications. Strategies that focus on providing routine vaccination to persons at higher risk for influenza complications have long been recommended, although coverage among most of these groups remains low [4]. Concurrently, there is a need to increase seasonal vaccine use through clear immunization policies as a way to stimulate industry to boost production capacity [5].

During the 2009/10 season, the influenza pandemic led to reinforcement of influenza vaccination recommendations in European countries, including Portugal. The pandemic also led to renewed interest in influenza vaccination surveillance.

Continued annual monitoring is needed to determine the effects of vaccine supply, changes in influenza vaccination recommendations, changes in groups targeted for vaccination, and other factors, on vaccination coverage among adults and children [4]. Vaccine coverage rates constitute the basic measure for evaluation of public health programs designed to improve vaccination uptake and for estimation of how the vaccination program affects the rate of disease.

Circumstantial factors (e.g., personal reasons) may affect the rate of vaccination [6]. In the last 12 years three major pandemic threats, not all caused by influenza virus, affected public and healthcare professionals’ perceptions of the need for influenza vaccine (IV) uptake. These pandemic threats were severe acute respiratory syndrome (SARS) in 2003, “avian influenza” (influenza A (H5N1)) in 2005, and the recent influenza A (H1N1)2009 pandemic. The rapid worldwide dissemination of SARS in 2003 was a rehearsal for the next influenza pandemic [7]. In 2005, the World Health Organization (WHO) declared that the emergence and persistence of influenza A (H5N1) in birds and the associated human fatalities were a public health threat and fostered early response strategies to contain the pandemic [8]. In April 2009, the spread of novel swine flu origin influenza A (H1N1) prompted the declaration of a pandemic by WHO in June of the same year [9]. Whether this raised awareness had a positive or negative effect on IV uptake varies according to country and to situation [6,10,11].

Every year, based on surveillance of clinic and laboratory data provided by a global network of Influenza Surveillance Centers, the WHO recommends the composition of the vaccine that will be used the next season [12]. The General Directorate of Health (DGS) in Portugal issues an information guide to Ministry of Health and private sector doctors in September/October of each year. The guide describes vaccine specifications for the current season and indications for vaccine uptake. Each flu season, the main objective of the DGS has been to increase IV coverage in high risk groups (i.e., individuals >65 years old and patients with specific chronic diseases) and priority groups of health professionals [13,14]. Table 1 describes how the recommendations have changed over time.

**Table 1:** Recommendations for seasonal influenza vaccination in Portugal, 1998/99 to 2010/11 seasons

**Season**	**Recommendation for seasonal influenza vaccination**
1998/00	Not available
2001/02	Elderly
	Individuals (>6 months) with chronic diseases and high-risk conditions
2002/03	Elderly
	Individuals (>6 months) with chronic diseases and high-risk conditions
	Healthcare workers
	Contacts of persons at high risk
2003/04	Elderly
	Residents of institutions for the elderly and the disabled
	Individuals (>6 months) with chronic diseases and high-risk conditions, including pregnant women
	Homeless
	Healthcare workers
	Contacts of persons at high risk
2004/05	Elderly
	Residents of institutions for the elderly and the disabled
	Individuals (>6 months) with chronic diseases and high-risk conditions, including pregnant women
	Homeless
	Healthcare workers
	Contacts of persons at high risk
2005/06	Elderly
	Residents of institutions for the elderly and the disabled
	Individuals (>6 months) with chronic diseases and high-risk conditions, including pregnant women
	Homeless
	Healthcare workers
	Contacts of persons at high risk
	Women in the 2^nd^ and 3^rd^ trimester of pregnancy
	Professionals that may be involved in culling poultry infected with influenza virus
2006/07	Elderly
	Residents of institutions for the elderly and the disabled
	Individuals (>6 months) with chronic diseases and high-risk conditions, including pregnant women
	Healthcare workers
	Contacts of persons at high risk
	Women in the 2^nd^ and 3^rd^ trimester of pregnancy
	Professionals that may be involved in culling poultry infected with influenza virus
2007/08	Elderly
	Residents of institutions for the elderly and the disabled
	Individuals (>6 months) with chronic diseases and high-risk conditions, including pregnant women
	Healthcare workers
	Contacts of persons at high risk
	Women in the 2^nd^ and 3^rd^ trimester of pregnancy
	Professionals that may be involved in culling poultry infected with avian influenza virus
2008/09	Elderly
	Residents of institutions for the elderly and the disabled
	Individuals (>6 months) with chronic diseases and high-risk conditions, including pregnant women
	Healthcare workers
	Contacts of persons at high risk
	Women in the 2^nd^ and 3^rd^ trimester of pregnancy
	Professionals that may be involved in culling poultry infected with avian influenza virus
2009/10^*^	Elderly
	Residents of institutions for the elderly and the disabled
	Individuals (>6 months) with chronic diseases and high-risk conditions, including pregnant women
	Healthcare workers
	Contacts of persons at high risk
	Women in the 2^nd^ and 3^rd^ trimester of pregnancy
2010/11	Elderly
	Residents of institutions for the elderly and the disabled
	Individuals (>6 months) with chronic diseases and high-risk conditions, including pregnant women
	Healthcare workers
	Contacts of persons at high risk
	Women in the 2^nd^ and 3^rd^ trimester of pregnancy

*Source:* General-Directorate of Health, Recommendations for seasonal influenza vaccine, Information Guide, 2000 to 2010.

^*^In 2009/10, a specific recommendation for vaccination against pandemic influenza virus A (H1N1) 2009 was also issued, and included the abovementioned groups (except elderly and residents of institutions) and also healthy children under 12 years old, caretakers of infants under 6 months old, and professionals performing core roles, according to priority groups.

In 2004, WHO established a 75% IV coverage target in the elderly (>65 years) that was in effect until 2010 [12]. In 2006, DGS established an interim target of 50% vaccine coverage among individuals aged 65 years and over for the 2006/07 season [14].

Despite the occurrence of a pandemic due to a new strain of influenza virus in 2009, the DGS has maintained the same seasonal IV recommendations for the major risk groups [15]. Concurrently, the recommendation expanded to include vaccination against pandemic influenza A (H1N1) 2009 for children from 6 months to 12 years. Older individuals were included only if they had a specific chronic disease [16].

Since the 1998/99 season, the Department of Epidemiology (DEP) of the National Institute of Health Doctor Ricardo Jorge (INSA; previously the National Health Observatory (ONSA)) has monitored IV coverage. This system is the only one in the country that estimates IV coverage in the general Portuguese population and in subgroups. This information cannot be obtained from a count of vaccine sales or from vaccine administration data.

This article describes national trends in seasonal IV coverage in Portugal from 1998 and 2010 in the general population and in age groups presents an analysis of progress towards WHO 2010 coverage goals. It also addresses the effects of the major pandemic threats (SARS in 2003, influenza A (H5N1) in 2005, and the influenza A (H1N1)2009 pandemic) on vaccination trends for the last 12 years.

## Methods

Between 1998/99 and 2010/11, INSA conducted 12 household telephone surveys using a panel of families (ECOS – Em Casa Observamos Saúde/*Observing Health at Home*). These surveys were used to collect data on IV coverage in the mainland Portuguese population.

The ECOS panel consisted of a random sample of Portuguese families with a landline telephone and of families with landline and mobile phone since the 2009/10 sample (dual sample frame). The sample was stratified and was evenly distributed to represent the five health regions of the country. Landline phone households were selected by simple random selection from the national telephone directory. Mobile phone households were selected by random digit generation. All households received a letter from INSA with an invitation to participate in the ECOS panel and provide informed consent. Telephone contact was then used to formalize participation and record each household member’s demographic data. The households included in each panel were renewed approximately every 3 years. The ECOS panel of families was approved by the Portuguese Data Protection Authority, which is in charge of ethical issues and protection of individual data collection in Portugal.

The seasons included in the surveys were the winters from 1998/99 to 2010/11, except for the winter of 2000/01. No investigation was carried out in 2000/2001 season owing to lack of financing.

All surveys used the same questionnaire. The questionnaire was presented to one individual (≥18 years of age) in each household using CATI (Computer Assisted Telephone Interview) technology. This individual provided information on his/her vaccination status and information on the household. The terminology “percent of vaccinated” used in reporting results refers to individuals who reported being vaccinated or on which the respondent said they were vaccinated.

The 1998/99, 1999/00, and 2001/02 surveys used the sample set formed in 1998. The surveys from 2002/03 to 2005/06 were carried out with a sample that was selected in 2002. The investigation in the 2006/07, 2007/08, and 2008/09 seasons was conducted with a sample formed in 2006. The 2009/10 and the 2010/11 surveys used a sample that was selected in 2010 (Table 2).

**Table 2:** **ECOS (Em Casa Observamos Saúde/*Observing Health at Home*) samples survey completion dates**

**Survey/season**	**Year ECOS sample formed**	**Completion date**
1998/1999	1998	May 1999
1999/2000	1998	February 2000
2001/2002	1998	July 2002
2002/2003	2002	May 2003
2003/2004	2002	March 2004
2004/2005	2002	April 2005
2005/2006	2002	May 2006
2006/2007	2006	February 2007
2007/2008	2006	February 2008
2008/2009	2006	January 2009
2009/2010	2010	April 2010
2010/2011	2010	February 2011

A detailed description of the ECOS methodology can be found in a published report [17]. Permission to use the data for this study was obtained from the National Institute of Health.

IV coverage was analyzed for the entire sample and for specific groups defined by age and sex. Information on demographic questions was collected during the initial survey done at household recruitment time.

For the surveys conducted in 1998/99 to 2008/09, all IV coverage estimates were adjusted by health region using the 2001 Portuguese population census data (2001 Census data from the National Institute of Statistics). For the surveys of the 2009/10 and 2010/11 periods that were conducted using the dual sample frame, the IV coverage estimates were adjusted by health region (Census population 2001) and for cell phone and landline phone coverage of Portuguese households using the methodology described in *Brick*[18] and *Kennedy*[19]. Weighting factors were adjusted by post stratification for population age and sex distribution.

To test the association (or independence) with disaggregation variables, we used the modified F-statistics of the second order adjustment of the Rao-Scott Chi-squared test [20] whose properties are presented in *Rao and Thomas*[21]. A 5% significance level was used for the statistical tests, and the null hypothesis was rejected when the probability of test significance (p-value) was <0.05. We also calculated 95% confidence intervals for all proportions.

We used a meta-regression model to test the linear trend in IV coverage throughout the study period, and the effect of the SARS, A (H5N1), and A (H1N1)2009 pandemics. Each survey estimate was weighted by the inverse of the variance in the logit scale. The model fitted to the logit of IV coverage included three dummy variables (one for each event) and a sequence of numbers from 1 to 12 years to measure and test the time trend effect. The model was adjusted to IV coverage in the general population and age groups.

All analyses were performed using the statistical programs SPSS [22] or STATA SE [23].

## Results

### Study samples

Total sample sizes varied between the minimum value of 2192 individuals (2008/09) and the maximum value of 4167 individuals (2001/2002).

For sex distribution, there were no significant deviations from the 2001 population census distribution (i.e., all confidence intervals included the census population estimates). There were small differences between the age group distribution in the panel data and the population age group distribution observed in the 2001 census. These differences varied by year.

### Influenza vaccine coverage from 1998/99 to 2010/11

**Figure 1:**
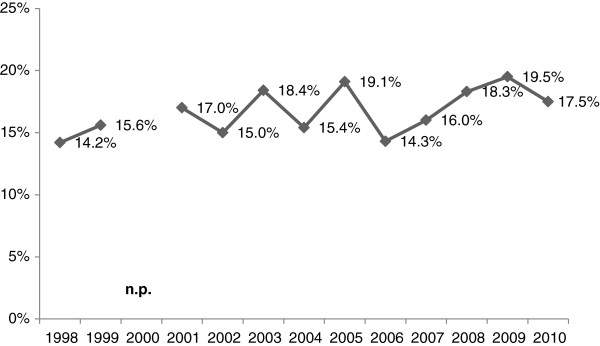
**Influenza vaccine coverage in Portugal.** Notes: n.p. not performed.

Between 1998/99 and 2010/11, IV coverage in the Portuguese general population (Table 3, Figure 1) varied between 14.2% (CI _95%_: 11.6%–16.8%) and 17.5% (CI _95%_: 17.6%–21.6%).

**Table 3:** Influenza vaccine coverage in Portugal for the 1998/99 to the 2010/11 seasons

	**1998/99**	**1999/00**	**2001/02**	**2002/03**	**2003/04**	**2004/05**	**2005/06**	**2006/07**	**2007/08**	**2008/09**	**2009/10**	**2010/11**
Total (n)	n = 2923	n = 3796	n = 4148	n = 2715	n = 2598	n = 2525	n = 2206	n = 2630	n = 2537	n = 2192	n = 2809	n = 2684
**Proportion vaccinated (%)**	14.2	15.6	17.0	15.0	18.4	15.4	19.1	14.3	16.0	18.3	19.5	17.5
(95% CI)	(11.6; 16.8)	(12.5; 18.7)	(14.5; 19.6)	(14.0; 16.0)	(16.7; 20.3)	(14.0; 17.0)	(17.4; 20.9)	(13.0; 15.8)	(14.5; 17.6)	(16.6; 20.1)	(17.6; 21.6)	(15.1; 20.3)

*Notes:* CI Confidence Interval; n sample size.

The lowest vaccine uptake occurred in 1998/99 and 2006/07 (14.2%, CI _95%_: 11.6%–16.8%; 14.3%, CI _95%_: 13.0%–15.8%), and the highest vaccine uptake occurred in the 2005/06 and 2009/10 seasons (19.1%, CI _95%_: 17.4%–20.9%; 19.5%, CI _95%_: 17.6%–21.6%).

There was no trend in IV coverage of the general population during the study period (p = 0.097).

### Influenza vaccine coverage by sex and age group

Over all the studied seasons, there were no statistically significant differences in IV coverage between women and men. However, IV coverage was higher for women than for men in 11 of the 12 seasons (Table 4). The difference between women and men was marginally significant for the 2003/04 (p = 0.058) and the 2010/11 (p = 0.056) seasons.

**Table 4:** Influenza vaccine coverage in Portugal for the 1998/99 to 2010/11 seasons, by sex and age

	**1998/99**	**1999/00**	**2001/02**	**2002/03**	**2003/04**	**2004/05**	**2005/06**	**2006/07**	**2007/08**	**2008/09**	**2009/10**	**2010/11**
**Sex** (%)	n = 1429	n = 1844	n = 1983	n = 1295	n = 1238	n = 1170	n = 1045	n = 1248	n = 1186	n = 1035	n = 1355	n = 1310
Male	13.9	15.5	16.9	15.0	16.6	14.8	19.2	13.0	14.9	16.7	19.1	15.9
	(11.9; 16.3)*	(13.6; 17.6)	(15.0; 19.0)	(12.7; 17.5)	(14.3; 19.3)	(12.8; 17.1)	(16.7; 21.9)	(11.1; 15.1)	(12.9; 17.2)	(14.5; 19.3)	(16.4; 22.1)	(13.1; 19.2)
	n = 1424	n = 1952	n = 2165	n = 1420	n = 1360	n = 1295	n = 1161	n = 1382	n = 1351	n = 1157	n = 1454	n = 1374
Female	14.4	15.7	17.1	14.9	20.1	15.9	18.9	15.5	16.9	19.7	20.0	19.1
	(12.3; 16.7)	(13.9; 17.8)	(15.3; 19.1)	(12.8; 17.3)	(17.7; 22.8)	(13.9; 18.1)	(16.7; 21.4)	(13.6; 17.7)	(14.9; 19.1)	(17.3; 22.2)	(17.2; 23.0)	(16.1; 22.5)
*p*^*†*^	*0.794*	*0.870*	*0.892*	*0.973*	*0.058*	*0.472*	*0.899*	*0.076*	*0.207*	*0.093*	*0.664*	*0.056*
**Age group** (%)	n = 390	n = 449	n = 481	n = 294	n = 290	n = 275	n = 214	n = 398	n = 352	n = 309	n = 457	n = 419
<15	12.1	14.6	10.2	5.7	8.2	6.1	11.3	4.4	3.9	5.5	12.9	9.6
	(8.7; 16.6)	(10.9; 19.1)	(7.3; 14.0)	(3.4; 9.4)	(5.2; 12.8)	(3.8; 9.7)	(7.4; 16.7)	(2.7; 7.0)	(15.3; 21.9)	(3.5; 8.8)	(9.3; 17.6)	(5.6; 16.3)
	n = 1266	n = 1570	n = 1617	n = 960	n = 936	n = 835	n = 704	n = 982	n = 944	n = 772	n = 1184	n = 1090
15-44	9.9	8.1	9.5	6.6	7.6	4.3	7.7	6.1	7.8	8.3	10.3	7.4
	(8.0; 12.1)	(6.6; 9.8)	(7.9; 11.4)	(4.9; 8.8)	(5.8; 9.8)	(3.1; 5.9)	(5.9; 10.2)	(4.7; 7.9)	(6.1; 9.8)	(6.5; 10.6)	(8.1; 12.9)	(5.1; 10.5)
	n = 818	n = 1066	n = 1244	n = 780	n = 810	n = 754	n = 707	n = 738	n = 776	n = 708	n = 800	n = 777
45 - 64	13.5	12.5	15.0	14.2	16.7	14.3	18.1	14.7	13.8	17.1	15.5	17.0
	(10.9; 16.6)	(10.3; 15.1)	(12.8; 17.5)	(11.4; 17.5)	(13.8; 20.0)	(11.8; 17.2)	(15.2; 21.4)	(12.2; 17.6)	(11.4; 16.6)	(14.3; 20.3)	(12.4; 19.1)	(13.5;21.2)
	n = 433	n = 616	n = 716	n = 523	n = 561	n = 601	n = 514	n = 355	n = 431	n = 396	n = 368	n = 398
≥65	31.3	39.0	41.9	36.9	46.9	39.0	41.6	50.4	51.0	53.3	52.2	48.3
	(26.1; 36.9)	(34.3; 43.8)	(37.6; 46.3)	(31.9; 42.3)	(41.9; 52.1)	(34.9; 43.3)	(37.1; 46.3)	(44.8; 55.9)	(45.8; 56.1)	(47.9; 58.6)	(45.6; 58.7)	(40.9; 55.7)
*p*	*<0.001*	*<0.001*	*<0.001*	*<0.001*	*<0.001*	*<0.001*	*<0.001*	*<0.001*	*<0.001*	*<0.001*	*<0.001*	*<0.001*

*Notes:* *95% confidence Interval; ^*†*^p-value; *n* sample size.

**Figure 2:**
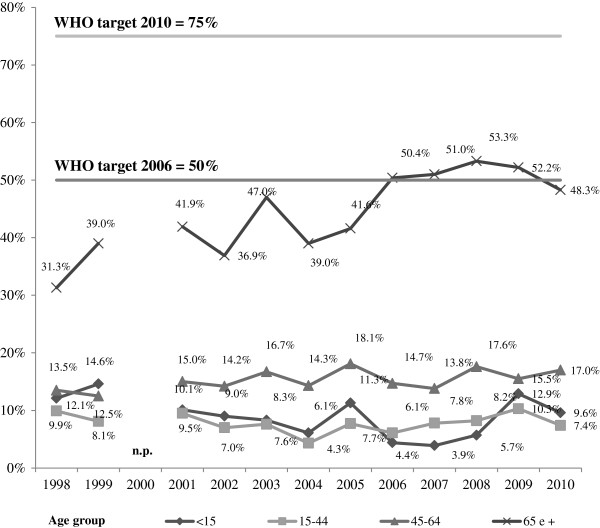
**Influenza vaccine coverage in Portugal by age group.** Notes: n.p. not performed.

Differences in IV coverage by age group occurred in all seasons (Table 4, Figure 2). In the younger age group (<15 years), a declining trend (with small fluctuations) was present from 1999/00 to 2008/09 (p = 0.005). This trend reversed in 2009/10, with one of the highest seasonal IV coverages for this age group (12.9%, CI _95%_: 9.3%–17.6%).

As expected, the highest IV coverage was observed in individuals >65 years old. Between 1998/99 and 2010/11, there was a gradual and significant increase in seasonal IV coverage in the elderly (p for trend <0.001).

In the 1998/99 season, IV coverage was 31.3% (CI _95%_: 26.1%**–**36.9%) and in the 2010/11 season it was 48.3%, which was similar to the 50% coverage achieved in the four previous seasons (Figure 2, Table 4).

An increasing trend was also identified in individuals aged 45–64 years old (p for trend 0.035). A trend was not present in the 15–44-year age group (p for trend 0.691).

### Impact of major pandemic threats on seasonal influenza vaccine coverage from 1998/99 to 2010/11

A meta-regression model was used to test for a potential effect on IV coverage for the seasons 2003/04, 2005/06, and 2009/10, while adjusting for the baseline vaccine coverage trend (Table 5). There was significantly higher coverage in the general population for two seasons: 2003/04 (p = 0.032) and 2005/06 (p = 0.018). Coverage in the 2009/10 season was not significant (p = 0.084).

**Table 5:** Inverse variance-weighted regression of vaccine coverage in the general population and by age group

		**All ages**	**0-14**	**15-44**	**45-64**	**65+**
	**Variable**	**Coef.±SE**	**Coef.±SE**	**Coef.±SE**	**Coef.±SE**	**Coef.±SE**
**Trend model**	Constant	-1.75 ± 0.09	-2.08 ± 0.29	-2.42 ± 0.16	-1.87 ± 0.08	-0.70 ± 0.01
		*p<0.001*	*p<0.001*	*p<0.001*	*p<0.001*	*p<0.001*
	Trend	0.02 ± 0.01	-0.05 ± 0.04	-0.01 ± 0.02	-0.02 ± 0.01	-0.06 ± 0.01
		*p=0.097*	*p=0.210*	*p=0.609*	*p=0.035*	*p<0.001*
**Trend and awareness events model**	Constant	-1.79 ± 0.08	-1.96 ± 0.25	-2.37 ± 0.17	-1.91 ± 0.08	-0.72 ± 0.11
		*p<0.001*	*p<0.001*	*p<0.001*	*p<0.001*	*p<0.001*
	Trend	0.02 ± 0.01	-0.08 ± 0.03	-0.02 ± 0.02	0.02 ± 0.01	0.07 ± 0.01
		*p=0.106*	*p=0.012*	*p=0.320*	*p=0.037*	*p<0.001*
	SARS 2003-2004	0.04 ± 0.10	0.04 ± 0.42	0.00 ± 0.28	0.17 ± 0.15	0.19 ± 0.17
		*p=0.032*	*p=0.930*	*p=0.999*	*p=0.044*	*p=0.256*
	A (H1N5) 2005-2006	0.22 ± 0.09	0.56 ± 0.42	0.06 ± 0.28	0.23 ± 0.11	-0.15 ± 0.17
		*p=0.018*	*p=0.180*	*p=0.833*	*p=0.044*	*p=0.368*
	A (H1N1) 2009-2010	0.18 ± 0.11	1.04 ± 0.43	0.47 ± 0.30	-0.04 ± 0.15	0.01 ± 0.23
		*p=0.084*	*p=0.015*	*p=0.116*	*p=0.783*	*p=0.954*

*Notes:* Coef.: inverse variance-weighted regression coefficients; SE: regression coefficients standard error; p-value tests the significance of regression coefficients.

Two coverage levels were significantly higher at the 5% confidence level: the IV uptake for the 45–64-year age group in the 2005/06 season (p = 0.044), and the vaccine uptake for the 0–14-year age group in the 2009/10 season (p = 0.015).

## Discussion

### Influenza vaccine coverage from 1998/99 to 2010/11

Between 1998/99 and 2010/11, IV coverage in Portugal varied between 14.2% and 17.5%. There was no consistent increase in percent IV uptake in the general population (p for trend 0.097).

During this time period (Figure 1, Table 3), there were four seasons that had notably higher IV coverage estimates: 2003/04, 2005/06, 2008/09, and 2009/10**.** However, except for 2003/04, these seasons did not have the highest coverage proportions in the elderly segment of the population (≥65 years), for whom vaccination has been long recommended. The seasonal IV coverage increase in the general population was primarily due to an increase in coverage in the 45–64-year age group in the 2005/06 and 2008/09 seasons and to an increase in the <15-year age group in the 2009/10 season (Table 4).

During the previous 12 years, healthy children and adolescents were not recommended risk groups for IV uptake in Portugal, except for during the 2009/10 influenza pandemic. Therefore, seasonal IV coverage levels among individuals <15 years old remained low. For most of the seasons, it was <10%. There were four seasons with slightly higher coverage levels in this age group: the 1998/99, 1999/00, 2005/06, and 2009/10 seasons, with coverage point estimates of 12.1%, 14.6%, 11.3%, and 12.9%, respectively.

Children are not considered to be a risk group for seasonal influenza. However, the general increased awareness related to the A (H1N1)2009 pandemic, and specific recommendations from Portuguese health authorities to vaccinate children may have affected seasonal vaccine uptake. More investigation is needed to address this hypothesis. However, the potential effect was not particularly strong considering that elevated vaccine uptake in season 2009/10 was not significantly higher than the other three seasons.

Individuals >65 years old had the highest coverage in all seasons. Values ranged from 31.3% in 1998/99 to 53.3% in the 2008/09 season (point estimates).

Regarding the 12-year pattern, a statistically significant increasing trend (p < 0.001) was observed for IV coverage in Portugal since 1998 in the elderly. However, after 50% coverage was achieved in 2006/07, a plateau was reached and no further improvement was observed in the last 4 years of the study period. The 2009 pandemic did not affect seasonal vaccination coverage in the elderly population in Portugal. Coverage was around 50%, which was similar to the three previous periods. This result is consistent with the fact that seasonal vaccine recommendations did not change throughout this period. Unchanged seasonal coverage rates were also observed in an elderly population in France [24].

In Europe, estimates for IV coverage in the elderly indicate that some countries failed to meet the target of 50% coverage in 2006. Portugal was not one of these countries [25]. However, other countries achieved the 75% coverage target for 2010. These countries were England, Scotland, Wales (79% in the 2005/06 season) [25], and The Netherlands (74% CI _95%_: 71%–77% in 2001/02) [26]. A recent study of 11 European countries (2006/07 season) found that Spain, with 71% coverage, was close to meeting the goal [27]. This study also estimated that there was 53% IV coverage in Portugal for individuals ≥65 years, which is consistent with the estimate obtained in our study (50.4%, CI _95%_: 44.8%–55, 9%). Compared with the other 10 countries included in the study (United Kingdom, Germany, Italy, France, Spain, Austria, Czech Republic, Ireland, Finland, and Poland), IV coverage in the elderly (≥ 65 years) in Portugal was sixth, but was similar to the overall estimate for all countries (53.2%).

In another European study that included 22 European countries (2006/07 season), Portugal ranked thirteenth for IV coverage in the elderly, and represented the median value for coverage in Europe [28]. This finding was consistent with the findings of the previous study.

Given that the elderly had been targeted for vaccination for many years and are the largest risk group, these results indicate that progress has been made toward meeting the WHO coverage goals. They reflect positively on Portuguese vaccination policies. Nevertheless, the 2010 75% coverage goal for 2010 remained far from being achieved. The 50% coverage plateau since 2006/07 indicates that additional effort is needed to further improve vaccination levels. Receiving advice from the family doctor/nurse has been identified as the main motivation to get vaccinated among the Portuguese population [29], followed (at a considerable distance) by old age as a reason. This result suggests that efforts directed at family doctors could have the greatest effect on IV uptake in the elderly. Public information campaigns that are directed at risk groups may also have an effect.

### Effect of major pandemic threats on influenza vaccine coverage from 1998/99 to 2010/11

We hypothesized that 2003/04, 2005/06, and 2009/10 were seasons during which there was special awareness about vaccination among the general Portuguese population, and/or among subgroups defined by age. We used a meta-regression model to test this hypothesis. These seasons were selected based on major public health threats worldwide: SARS in 2003/04, the increase in H5N1 virus infections in humans in Southeast Asia in 2005/06, and the declaration of an influenza pandemic in 2009/10.

Global and national health authorities, and the medical community worldwide, reinforced flu vaccination recommendations in 2003/04 [30]. In 2005/06, the pandemic threat, with associated vaccination recommendations, led to news and opinions that circulated in the medical community and in the general population. This news coverage could have led to an increased demand for the influenza vaccine. In fact, in a study on IV coverage in Germany (2001–2006), the authors found that coverage increased during the 2005/06 season. They suggested that the greater media focus on pandemic influenza was one factor that explained the increased vaccine demand [31].

In 2009/2010, the declaration of a worldwide influenza pandemic by WHO focused the attention of the media on influenza. Health ministries worldwide, including in Portugal, issued broad guidance to health services that was also reported by the media.

The results of the meta-regression model (Table 5) indicate that in the general population, influenza coverage was significantly greater in two seasons, 2003/2004 (p = 0.032) and 2005/2006 (p = 0.018). The seasonal influenza coverage in the general population during the 2009/2010 season was not significantly greater (p = 0.084). However, an examination of IV coverage differences for these three seasons, according to the age group, revealed that two coverage estimates were significant: the increase in vaccine uptake of the 45–64 segment of the population in the 2005/06 season (p = 0.044) and the increase in IV uptake in the 0–14 group in the 2009/10 season (p = 0.015).

These findings indicate that when accounting for the baseline trend, there was an increase in IV coverage during the 2003/04 and 2005/06 seasons, which suggests that awareness about vaccination could have increased in the general population and in specific age groups.

Although the 12.9% coverage in children was not the highest of all the seasons was still an important increase. Risk perception regarding flu in children may have increased during the 2009/10 season, and pandemic awareness and special recommendations for children to be vaccinated during the pandemic could have affected seasonal vaccine uptake in Portugal in the 2009/10 season in this group.

Risk perception affects vaccine uptake [32,33]. Although results vary in degree and direction, perceptions about the risk of disease and severity of infection that follow major pandemic threats may affect uptake of seasonal influenza vaccine [33-35]. The results of our study suggest that these events may have had a positive effect on IV uptake in Portugal. More research is needed to understand the factors underlying an individual’s decision to be vaccinated against influenza.

### Limitation of the study

ECOS is a sample of families from mainland Portugal, with landline and mobile telephones, who agree to complete periodic health surveys. Residents of Portugal who do not have a landline or mobile telephone were not represented in this study.

Between 1998/1999 and 2010/2011, the ECOS panel sample was renewed three times (2002, 2006, and 2010). Therefore, the estimates of IV coverage were obtained using four different samples that were selected using the same methodology. Using the same sample for more than one consecutive season could lead to biased coverage estimates. Specifically, the application of the questionnaire to the same sample in two consecutive seasons could lead to a greater proportion of individuals who choose to be vaccinated the next season. This change in behavior could artificially increase IV coverage, which would not represent coverage in the general population. There is no evidence of this bias in our study, because in each population group, IV coverage increase was not consistent or systematic for the four periods between changes in the sample population (1998 to 2001, 2002 to 2005, 2006 to 2009, and 2010 to the present).

As previously described, the representativeness of the samples studied in comparison with estimates from the 2001 census of the Portuguese mainland population found age deviations from this reference population. These deviations could be translated into an IV coverage bias in the general population. However, in risk groups for whom vaccination is recommended, particularly the elderly (≥ 65 years), and for whom IV coverage monitoring is more critical for control measures, the age bias presented by our samples was less relevant.

Recall bias may occur when individual recall of information is used to obtain data. However, individuals were vaccinated some months before each survey and this time differed from survey to survey. Additionally, only one individual (>18 years) per household answered questions about vaccination status of household members. Surveying all of the individuals in a household would make the procedure more complex and could lead to a failure of the entire process, which occurred during previous surveys using the ECOS panel [17].

## Conclusions

From 1998/99 to 2010/11, seasonal IV coverage in Portugal varied between 14.2% and 17.5%. No significant increasing trend was observed in the Portuguese general population during this period.

There was a clear, increasing trend in IV coverage in the elderly population (p < 0.001). Coverage increased from 31.3% in 1998/99 to 48.3% in 2010/11. After 2006/07, however, IV coverage in the elderly has remained near 50%, which suggests that there has been a slowdown in the growth trend. Thus, in 2010, the 75% WHO target in this major risk group had not been met. This result indicates that there is a need to improve the influenza vaccination strategy in Portugal to comply with the ambitious coverage goals proposed by WHO.

The major pandemic threats of the last decade had an effect on seasonal influenza vaccination. There was a significant increase in vaccine uptake in the general population in 2003/04 (p = 0.032) and in 2005/06 (p = 0.018). The 2009/10 seasonal vaccine coverage in the general population was not significantly higher when accounting for the baseline trend, but the 12.9% of vaccination occurred in individuals <15 years old. Although not the highest of all the seasons, this result was significant when accounting for vaccination trend in this age group during the past decade.

## Competing interests

The authors declare that they have no competing interests.

## Authors’ contributions

CP participated in drafting the manuscript and in the statistical analysis. BN participated in the design and coordination of the study, in the statistical analysis, and reviewed the manuscript. MJ reviewed the manuscript and participated in its design and organization. All authors read and approved the final manuscript.

## Pre-publication history

The pre-publication history for this paper can be accessed here:

http://www.biomedcentral.com/1471-2458/13/1130/prepub
